# Bladder neck reconstruction in girls’ pelvic fracture bladder neck avulsion and urethral rupture

**DOI:** 10.1186/s12894-020-00741-z

**Published:** 2020-11-04

**Authors:** Rong Lv, Chongrui Jin, Huiquan Shu, Lin Wang, Yinglong Sa

**Affiliations:** 1 Department of Urology, Shanghai Jiao Tong University Affiliated Sixth People’s Hospital, Shanghai, China; 2 Department of Urology, Shanghai Children’s Hospital, Shanghai Jiao Tong University, Shanghai, China; 3 Shanghai Eastern Institute of Urologic Reconstruction, Shanghai, China

**Keywords:** Female pediatric urethral injury, Bladder neck reconstruction, Urinary continence, Pediatric pelvic trauma

## Abstract

**Background:**

Girls’ pelvic fracture bladder neck avulsion and urethral rupture is rare however it causes great morbidity. The management is complex and not standard yet. We report our experience and a technique of bladder neck reconstruction with anterior bladder wall flap.

**Methods:**

We retrospectively analysed data of 5 girls with pelvic fracture bladder neck avulsion and urethral rupture admitted to our institution from July 2017 to October 2019. They all came to our institution with a suprapubic tube. Patients’ trauma was all initially treated at other hospitals, 4 had suprapubic cystotomy and 1 had urethral realignment. One girl also had three other urethroplasties at other hospitals. We took pubectomy, posterior ureth roplasty and bladder neck reconstruction with anterior bladder wall flap in these 5 girls. Post-operative assessments included voiding cystourethrography, uroflowmetry and urethroscopy after urethral catheter removal. Verbal consent to participate was obtained from the parent or legal guardian of the children.

**Results:**

Operation time ranged from 120 to 180 min. Follow-up time is 12 to 27 months. Uroflowmetry showed that maximum urine flow rate improved significantly. Cystourethrography indicated good continuity of the urethra. Two girls had urinary incontinence postoperatively but were continent 3 months later. One patient developed vesical-abdominal fistula and got repaired by surgery 6 months later. She was continent ever since. Other complications were not observed during the follow-up period.

**Conclusions:**

Our method of bladder neck reconstruction using bladder flap as a patch is feasible and provides good continence, especially for those with serious bladder neck avulsion and urethral rupture caused by extensive trauma and those who had posttraumatic urethral distraction needed second repair.

## Background

Comparing to male urethra, female urethra with an average length of 4 cm is much shorter and less fixed in the pelvic, thus the chance of getting injured is low. Although pelvic fracture urethral and bladder injuries in girls are rare, the management is complex. Unlike male patients, there is no standard treatment for female pelvic fracture urethral injury and the surgical management in published literature remains controversial because the morbidity of postoperative incontinence and stricture were still high. Hence in this article, we introduced 5 cases of girls with intensive bladder neck avulsion and urethral rupture associated with pelvic fracture and elaborated our surgical technique of bladder flap bladder neck reconstruction.

## Methods

### Clinical materials

From July 2017 to October 2019, 5 girls with bladder neck avulsion and urethral rupture caused by traumatic pelvic fracture were admitted to our institution and accepted surgery. Their clinical characteristics were listed in Table 1. Their mean age is 7.4 years old. Trauma was caused by motor vehicle accident in 4 cases, pedestrian-car accident in 1 case. All the patients were initially treated at other hospitals when the trauma happened, hence the type and extent of urinary injury weren’t available. Their urinary injuries were dealt with suprapubic cystotomy in 4 and 1 had urethral realignment. Their pelvic fractures were managed with external fixation in 3 cases and internal fixation in 2 cases. The interval between the trauma and our surgery ranged from 6 months to 9 years. They all came to our institution with a suprapubic tube. One girl had posterior urethra end-to-end anastomosis, percutaneous nephrolithotomy and posterior urethroplasty using bladder mucosa at other hospitals.

**Table 1:** Clinical information of patients

	Age	Etiology	Condition history	Operation history	Complications
Case 1	13 years old	Motor vehicle accident	9 years	Suprapubic cystotomy, posterior urethra end-to-end anastomosis, percutaneous nephrolithotomy and posterior urethroplasty using bladder mucosa	None
Case 2	9 years old	Pedestrian-car accident	7 months	Urethral realignment	Vesicovaginal fistula; urinary incontinence
Case 3	8 years old	Motor vehicle accident	1 year	Suprapubic cystotomy	None
Case 4	21 months	Motor vehicle accident	6 months	Suprapubic cystotomy	None
Case 5	5 years old	Motor vehicle accident	2 years	Suprapubic cystotomy	None

### Preoperative evaluations

**Fig. 1:**
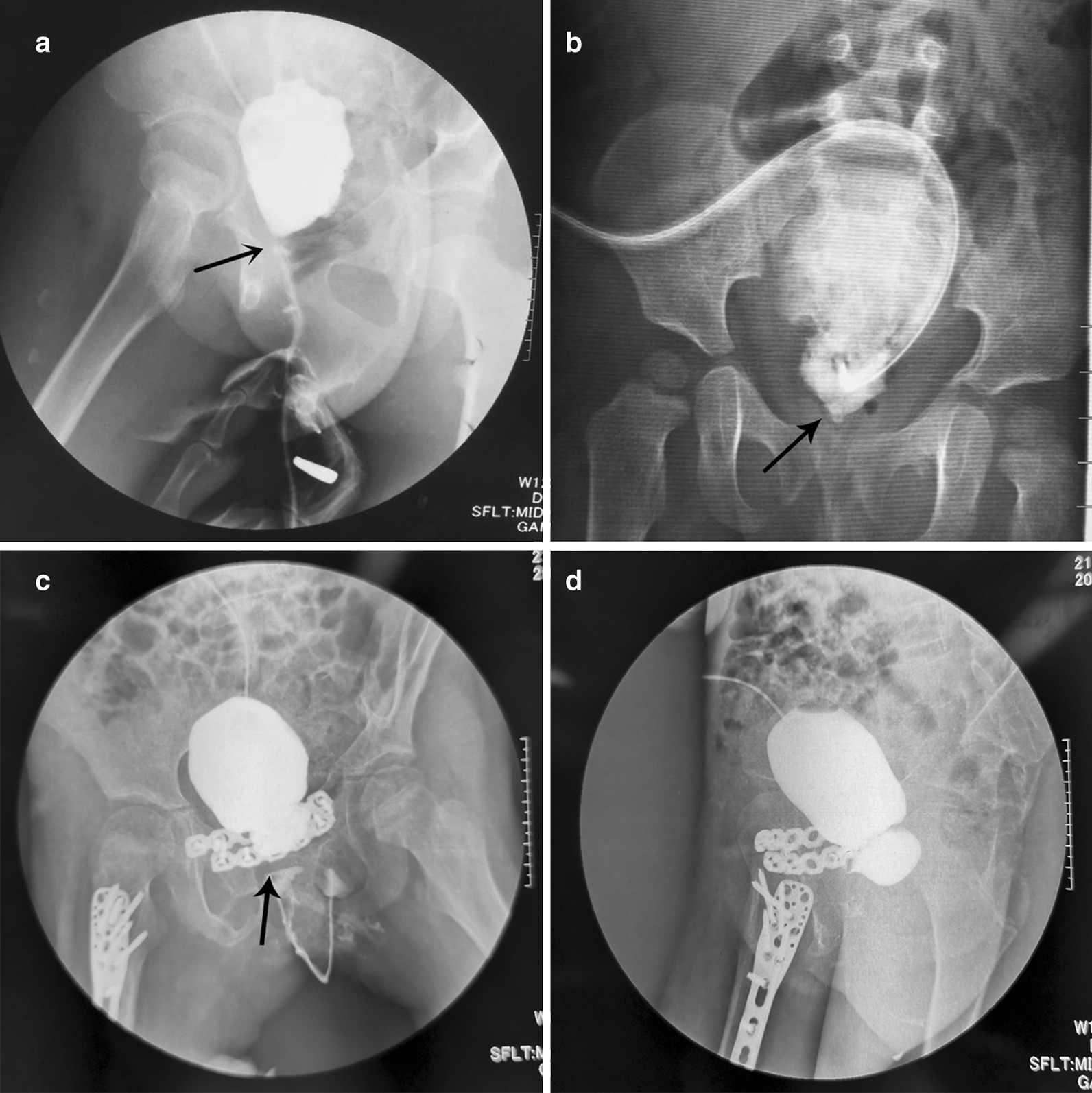
**a**–**c** Preoperative voiding cystourethrography showed completely atresia of bladder neck; **d** preoperative voiding cystourethrography showed vesicovaginal fistula

we evaluated patient medical history, physical examination and routine laboratory investigations before the operation. Blood urea and serum creatinine in patients were normal and USG abdomen showed bilateral normal kidneys. Voiding cystourethrography showed completely atresia of bladder neck and urethral distraction (Fig. 1a). Vesicovaginal fistula was found in one girl (Fig. 1c, d). Urethroscopy entered the urethra for around 2 cm and was blocked. Suprapubic cystoscopy showed the bladder neck was obliterate, ureteric orifices were normal and vesicovaginal fistula was found in one patient. Vaginoscopy under anesthesia was also done to rule out urethrogenital fistula.

### Surgical technique

**Fig. 2:**
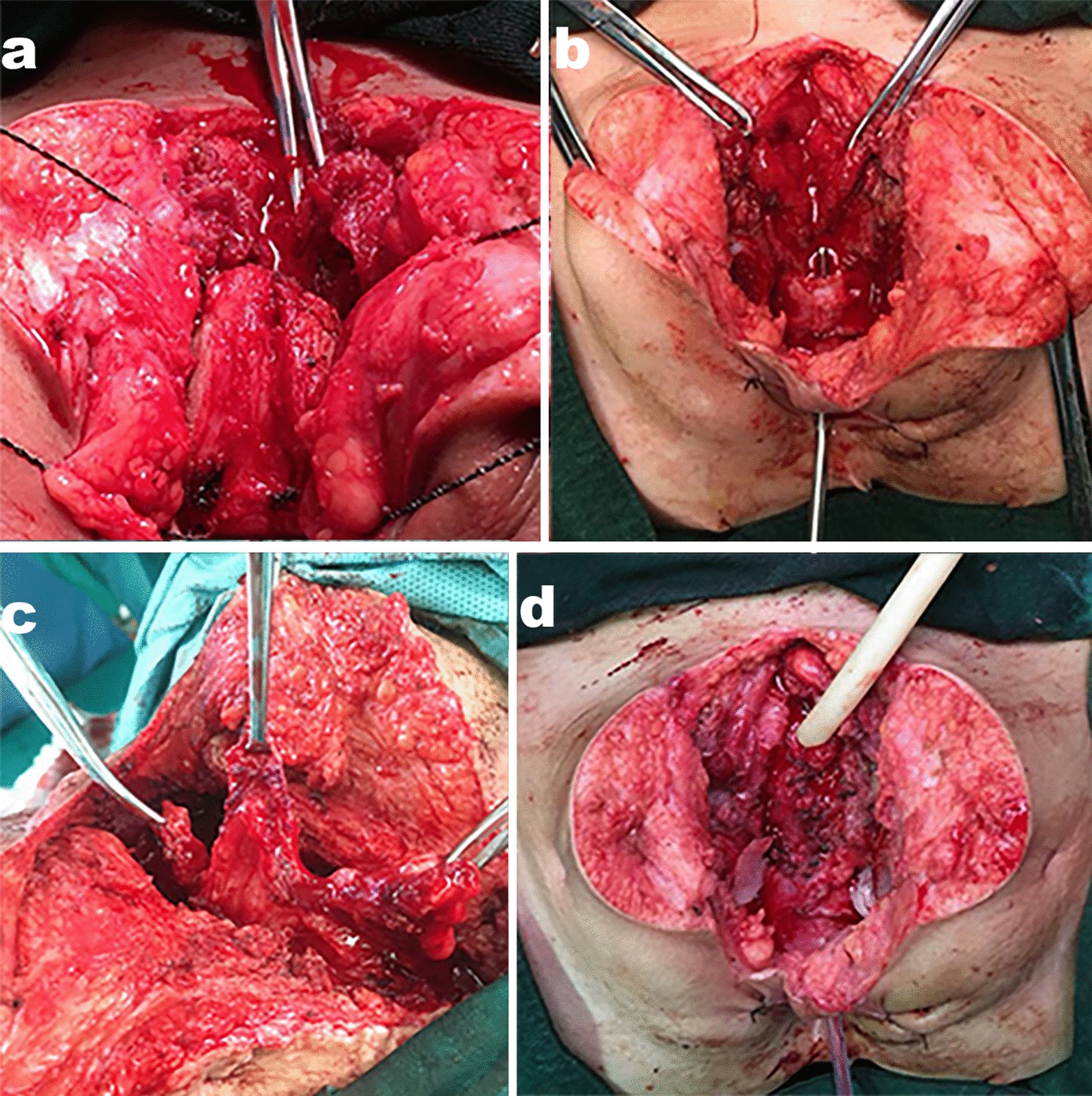
**a** Pubectomy with Gigli saw; **b** the defect between the atresic bladder neck and the obstructed proximal urethra is 1 cm; **c** acquiring the bladder flap from the anterior bladder wall; **d** reconstruction of the bladder neck and proximal urethra

All patients were administered by general anesthesia and were under lithotomy position. The surgery was accomplished by combined vaginal and transpubic approach through a lower midline abdominal incision. Then a 2 cm around segment of pubic bone was cut using a Gigli saw to get enough surgical field of the bladder neck and proximal urethra, then the atresic bladder neck which was obstructed by fibrosis was showed. Under the guidance of a urethral dilator, we could see that remain distal urethra length is 1–2 cm and the defect between the atresic bladder neck and the obstructed proximal urethra is 0.5–1.5 cm. The obstructed urethra and bladder neck were incised longitudinally under the guidance of urethral dilator and periurethral fibrous tissue was excised. The process of bladder neck reconstruction is rather like a modified Y–V plasty. A T-shaped incision was made at the anterior bladder wall so as to obtain two 1.5–3 cm long, 1–2 cm width well vascularized flaps. We reserved the dorsal part of bladder neck and proximal urethra. Then the bladder wall flaps are flipped downwards as a patch to augment the ventral part of bladder neck and proximal urethra. Fat tissues were wrapped around the new urethra and bladder neck to eliminate the cavity around the urethra. A 6–14 Fr silicone Foley catheter and a suprapubic catheter were placed after bladder closure (Fig. 2). A drainage tube was left in the pelvis.

### Postoperative management

Prophylactic antibiotics were used for 3 days. The pelvic drainage tube was removed 3 days after the surgery. The urethral silicone catheter was left indwelled for 3 weeks and the suprapubic catheter was removed 1 weeks after the removal of urethral catheter. Postoperative evaluations included voiding cystourethrography, uroflowmetry and urethroscopy after urethral catheter removal.

## Results

**Fig. 3:**
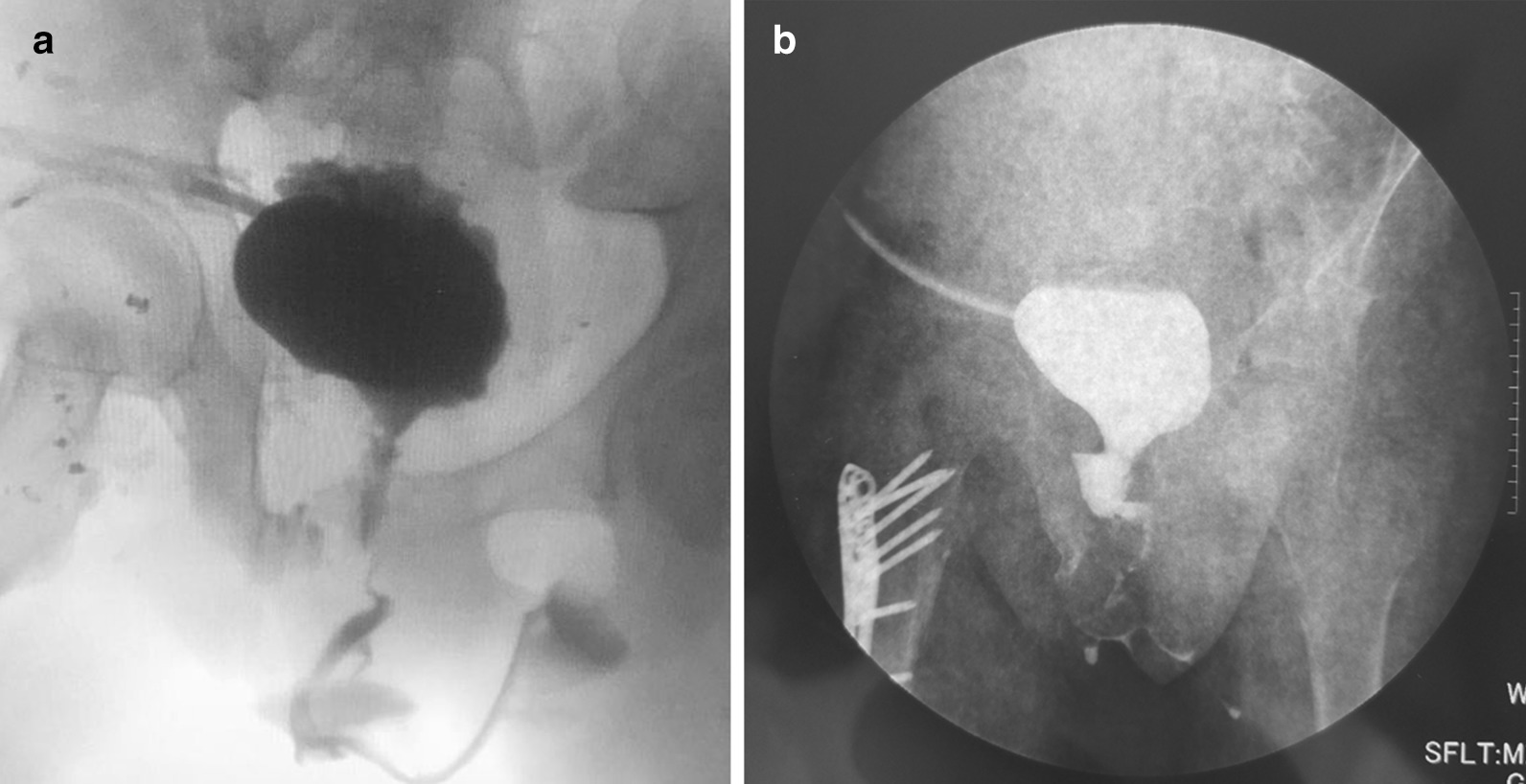
Post-operative voiding cystourethrography showed good continuity of urethra and a wide bladder neck

Operation time ranged from 120 to 180 min. Follow-up time is 12 to 27 months. Uroflowmetry showed that maximum urine flow rate improved significantly, ranging from 16.5 to 20 ml/s, and mean value is 17 ml/s. Cystourethrography indicated good continuity of the urethra (Fig. 3). Two girls had urinary incontinence postoperatively but were continent 3 months later. One patient developed vesical-abdominal fistula due to multiple surgical experiences and poor healing of abdominal incision. The fistula got repaired by surgery 6 months later and she was continent ever since. Other complications were not observed during the follow-up period.

## Discussion

Female urethral injuries caused by pelvic fracture is a rare occurrence because the female urethral is short and has a good mobility in the pelvic. As for children, the skeletally immature pelvis is more plastic and flexible because there is a more percentage of cartilage, a more porous cortical bone, and the pubic symphysis and sacroiliac joints are more elastic [1], hence the pelvic absorbs higher energy during trauma. Also, girl’s bladder neck and urethra are more vulnerable because the relative higher position in the pelvis [2], which may explain why the proportion of girls urethral injuries is higher than female adults in the current literature [3]. Although rare, the management of female pelvic fracture urethral injuries is complex and there is no guideline yet.

For the diagnosis of bladder and urethral injury in those patients, their genitourinary injuries were often ignored in the emergency room during the primary trauma, unless there were symptoms of hematuria and vaginal bleeding, cause patient’s vitals and life-threatening risks were put in the first place. Black et al. [4] report in their study that nearly 25% of these patients were diagnosed with urinary injuries accidentally during surgical exploration. Hence we recommend a careful physical examination in the emergency room, and unstable patients might need lower genitourinary tract imaging [5]. Even diagnosed, most patients were dealt with suprapubic cystotomy. In our cases, all the patients had suprapubic cystotomy after the trauma happened, preoperative cystourethrography showed bladder neck atresia and urethral distraction. We also advise that urethroscopy is needed to know the lesions of bladder and rule out genitourinary fistula. Because of partial healing and fibrosis of their urethral injury, the bladder neck is completely atresic, and the restoration of urinary tract continuity and continence became rather tricky.

Casselman et al. [6] reported the first case of traumatic complete disruption of the female membranous urethra in the English literature in 1977, the patient was a 2.5 years old girl with an approximately 1 cm avulsion of urethra at the urogenital diaphragm, Casselman drawn down the bladder and the urethra and anastomosed, but after 8 weeks the girl had urethral stricture and the problem was solved by urethral dilation. Direct anastomosis of the urethra would most likely lead to stricture, the missing part of urethra needs to be replaced. Vaginal flap and buccal mucosal graft urethroplasty were more reported and used for female urethral reconstruction, while these techniques are more suitable for female urethral stricture and small urethral loss. For long urethral defect or proximal urethral injuries bladder wall flaps can be adopted for urethroplasty [7–9].The female urethral replacement is mainly accomplished by anterior bladder wall (Tanagho) tube or tubularization of vaginal mucosa [10]. Tanagho was first to bring up the technique of reconstructing bladder neck with tubular anterior bladder flap in 1981 [11]. Nayyar et al. [12] reported their technique of a novel anterior bladder tube for the treatment of traumatic bladder neck contracture based on Tanagho technique. Their report included 3 female patients with good results, which also need further followup and more literature report. For surgical access, we took the combined vaginal and transpubic access, it is recognized that combined vaginal-partial transpubic access is a dependable method for female pediatric patients with complicated bladder neck and urethral trauma after pelvic fracture [13]. These pediatric female patients all came to our institution with suprapubic catheter, the intervals since their primary trauma are mostly over 6 months, the bladder neck is obliterated by scar tissues and the urethra is atresic, hence the transpubic access provides the maximum operation field and we took total pubectomy to get access to the bladder neck. With combined vaginal approach, the distal urethra is located and urethrovaginal fistulas were repaired. Through the T shape incision of the anterior bladder, two well-vascularized and free flaps are acquired. Giving that the fundus of bladder neck is generally existed in those patients, we used the bladder flap to augment the ventral part of bladder neck and then anastomosed it with distal urethra. Thus, the bladder neck and proximal urethra were expanded, decreasing the chance of further stricture.

Controversy also exist in the surgical repair timing. Black et al. [4] indicated that the bladder neck injury should be repaired primarily because it is crucial in continence. Patel et al. [3] did a systematically review of the literature about female urethral injuries associated with pelvic fracture, their results showed that patients who had primary alignment were more likely to have urethral stenosis and fistula. From our perspective, patients who had extensive urethra injury often accomplished by serious damage of other organs, the principle is to deal with life-threatening risks first, so the patients were mostly managed with suprapubic cystotomy and deferred repair [14].

The mechanism of female continence is not completely clarified yet. It is generally thought that the female urinary continence mechanism is mainly made of the urinary sphincter complex, bladder trigone and the pelvic floor. The urinary sphincter complex includes the inner smooth muscle layer and the external striated muscle layer. The smooth muscle layer mainly locates at the level of bladder neck and the external striated sphincter muscle covers from the bladder neck to proximal urethra. Hence the bladder neck is of great importance in urinary continence. The urethral smooth muscle is under sympathetic control while the striated muscle is mostly under voluntary control. The running of autonomic nerve fibers is near lateral vaginal walls. The innervation of striated muscle transverses the pelvic and enters the caudal third of the urethra laterally [15–21]. The bladder musculature has 3 different muscular layers and forms the internal urethral sphincter at bladder neck. Bladder and urethral muscles have β-adrenergic inhibitory receptors and α-adrenergic excitatory receptors, which can lead to the relaxation and contraction of these muscles [16, 22, 23]. The muscular distribution and neuroreceptors of bladder is thought to promote continence in bladder flap urethral reconstruction [11]. In Tanagho technique and existed studies adopting the method of bladder wall flap urethroplasty, the stenosis bladder neck was transected and the fibrous tissues were completely excised, which we think might sacrifice some function of the bladder neck [10, 11, 14, 24]. An intact bladder neck is crucial to continence, not only because it is where the detrusor locate at and it’s sphincteric, but also because it is where the autonomic nerve and somatic nerve converge. In our surgical procedure, we just incised the atresia bladder neck and used the bladder flap as a patch to expand it. More importantly the dorsal part of bladder neck was preserved, which we thought might favor for continence because more nerve fibers and the intactness of bladder neck might get saved in this way.

## Conclusions

Our method of bladder neck reconstruction using bladder flap as a patch is feasible and provides good continence, especially for those with serious bladder neck avulsion and urethral rupture caused by extensive trauma and those who had posttraumatic urethral distraction needed second repair.

## Data Availability

The datasets used during the current study are available from the corresponding author on reasonable request.
